# Social distancing and extremely preterm births in the initial COVID-19 pandemic period

**DOI:** 10.1038/s41372-024-01898-3

**Published:** 2024-02-22

**Authors:** Vivek V. Shukla, Benjamin A. Carper, Namasivayam Ambalavanan, Matthew A. Rysavy, Edward F. Bell, Abhik Das, Ravi M. Patel, Carl T. D’Angio, Kristi L. Watterberg, C. Michael Cotten, Stephanie L. Merhar, Myra H. Wyckoff, Pablo J. Sánchez, Neha Kumbhat, Waldemar A. Carlo, the Eunice Kennedy Shriver National Institute of Child Health and Human Development Neonatal Research Network, Richard A. Polin, Abbot R. Laptook, Martin Keszler, Angelita M. Hensman, Elisa Vieira, Lucille St. Pierre, Anna Maria Hibbs, Michele C. Walsh, Nancy S. Newman, Sarah Smucney, Arlene Zadell, Brenda B. Poindexter, Kurt Schibler, Cathy Grisby, Kristin Kirker, Sandra Wuertz, Juanita Dudley, Traci Beiersdorfer, Julia Thompson, Ronald N. Goldberg, Joanne Finkle, Kimberley A. Fisher, Matthew M. Laughon, Gennie Bose, Cindy Clark, Stephen D. Kicklighter, Donna White, David P. Carlton, Yvonne Loggins, Judith Laursen, Colleen Mackie, Diane I. Bottcher, Andrew A. Bremer, Rosemary D. Higgins, Stephanie Wilson Archer, Jon E. Tyson, Amir M. Khan, Barbara J. Stoll, Gabriela Dominguez, Elizabeth Eason, Donna J. Hall, Apoorva Mahatme, Karen Martin, Ilse Reyna, Emily K. Stephens, Jaleesa Wade, Michelle White, Leif D. Nelin, Sudarshan R. Jadcherla, Jonathan L. Slaughter, Patricia Luzader, Jacqueline McCool, Kyrstin Warnimont, Jessica Purnell, Kristi Small, Melanie Stein, Rox Ann Sullivan, Laura Marzac, Hallie Baugher, Eli Zettler, Bethany Miller, Demi R. Beckford, Brittany DeSantis, Rachel Reedy, Marie G. Gantz, Carla M. Bann, Kristin M. Zaterka-Baxter, Jenna Gabrio, David Leblond, Jeanette O’Donnell Auman, Krisa P. Van Meurs, David K. Stevenson, Valerie Y. Chock, M. Bethany Ball, Barbara P. Recine, Elizabeth N. Reichert, Monica V. Collins, Shirley S. Cosby, Tarah T. Colaizy, Heidi M. Harmon, Michelle L. Baack, Laurie A. Hogden, Karen J. Johnson, Mendi L. Schmelzel, Jacky R. Walker, Claire A. Goeke, Sarah E. Faruqui, Brenda J. Coulter, Bailey M. Schrimper, Syndney S. Jellison, Chelsey Elenkiwich, Megan M. Henning, Megan Broadbent, Sarah Van Muyden, Janell Fuller, Robin K. Ohls, Sandra Sundquist Beauman, Conra Backstrom Lacy, Mary Hanson, Elizabeth Kuan, Sara B. DeMauro, Eric C. Eichenwald, Soraya Abbasi, Christine Catts, Aasma S. Chaudhary, Megan A. Dhawan, Sarvin Ghavam, Toni Mancini, Karen M. Puopolo, Jonathan Snyder, Ronnie Guillet, Anne Marie Reynolds, Satyan Lakshminrusimha, Michael G. Sacilowski, Mary Rowan, Rosemary Jensen, Rachel Jones, Alison Kent, Diane Prinzing, Ann Marie Scorsone, Kyle Binion, Stephanie Guilford, Constance Orme, Premini Sabaratnam, Daisy Rochez, Emily Li, Jennifer Donato, Luc P. Brion, Joanne Duran, Frances Eubanks, Michelle Harrod, Pollieanna Sepulvida, Diana M. Vasil, Bradley A. Yoder, Mariana Baserga, Stephen D. Minton, Mark J. Sheffield, Carrie A. Rau, Susan Christensen, Kathleen Coleman, Jennifer O. Elmont, Barbara L. Francom, Jamie Jordan, Manndi C. Loertscher, Trisha Marchant, Earl Maxson, Kandace McGrath, Hena G. Mickelsen, D. Melody Parry, Katherine Tice, Kimberlee Weaver-Lewis, Kathryn D. Woodbury

**Affiliations:** 1 Division of Neonatology, University of Alabama at Birmingham, Birmingham, AL USA; 2 Social, Statistical, and Environmental Sciences Unit, RTI International, Research Triangle Park, NC USA; 3 Department of Pediatrics, McGovern Medical School at The University of Texas Health Science Center at Houston, Houston, TX USA; 4 Department of Pediatrics, University of Iowa, Iowa City, IA USA; 5 Social, Statistical and Environmental Sciences Unit, RTI International, Rockville, MD USA; 6 Emory University School of Medicine, Department of Pediatrics, Children’s Healthcare of Atlanta, Atlanta, GA USA; 7 University of Rochester School of Medicine and Dentistry, Rochester, NY USA; 8 University of New Mexico Health Sciences Center, Albuquerque, NM USA; 9 Department of Pediatrics, Duke University, Durham, NC USA; 10 Department of Pediatrics, University of Cincinnati College of Medicine, Cincinnati, OH USA; 11 Department of Pediatrics, University of Texas Southwestern Medical Center, Dallas, TX USA; 12 Department of Pediatrics, Nationwide Children’s Hospital, The Ohio State University College of Medicine, Columbus, OH USA; 13 Department of Pediatrics, Division of Neonatal and Developmental Medicine, Stanford University School of Medicine and Lucile Packard Children’s Hospital, Palo Alto, CA USA; 14 Division of Neonatology, College of Physicians and Surgeons, Columbia University, New York, NY USA; 15 Alpert Medical School of Brown University and Women & Infants Hospital of Rhode Island, Providence, RI USA; 16 Case Western Reserve University, Rainbow Babies & Children’s Hospital, Cleveland, OH USA; 17 Cincinnati Children’s Hospital Medical Center, University of Cincinnati Medical Center, and Good Samaritan Hospital, Cincinnati, OH USA; 18 Duke University School of Medicine, University Hospital, University of North Carolina, Duke Regional Hospital, and WakeMed Health and Hospitals, Durham, NC USA; 19 Emory University, Children’s Healthcare of Atlanta, Grady Memorial Hospital, and Emory University Hospital Midtown, Atlanta, GA USA; 20 Eunice Kennedy Shriver National Institute of Child Health and Human Development, Bethesda, MD USA; 21 McGovern Medical School at The University of Texas Health Science Center at Houston, Children’s Memorial Hermann Hospital, and Memorial Hermann Southwest Hospital, Houston, TX USA; 22 Nationwide Children’s Hospital, The Abigail Wexner Research Institute at Nationwide Children’s Hospital, Center for Perinatal Research, The Ohio State University College of Medicine, The Ohio State University Wexner Medical Center, and Riverside Methodist Hospital, Columbus, OH USA; 23 Stanford University and Lucile Packard Children’s Hospital, Palo Alto, CA USA; 24 University of Alabama at Birmingham Health System and Children’s Hospital of Alabama, Birmingham, AB USA; 25 University of Iowa and Sanford Health, Iowa city, IA USA; 26 University of Pennsylvania, Hospital of the University of Pennsylvania, Pennsylvania Hospital, and Children’s Hospital of Philadelphia, Philadelphia, PA USA; 27 University of Rochester Medical Center, Golisano Children’s Hospital, and the University at Buffalo John R. Oishei Children’s Hospital of Buffalo, Rochester, NY USA; 28 University of Texas Southwestern Medical Center, Parkland Health & Hospital System, and Children’s Medical Center Dallas, Dallas, TX USA; 29 University of Utah Medical Center, Intermountain Medical Center, McKay-Dee Hospital, Utah Valley Hospital, and Primary Children’s Medical Center, Provo, UT USA

**Keywords:** Epidemiology, Paediatrics

## Abstract

**Hypothesis:**

Increased social distancing was associated with a lower incidence of extremely preterm live births (EPLB) during the initial COVID-19 pandemic period.

**Study design:**

Prospective study at the NICHD Neonatal Research Network sites comparing EPLB (22^0/7^–28^6/7^ weeks) and extremely preterm intrapartum stillbirths (EPIS) rates during the pandemic period (March-July, weeks 9–30 of 2020) with the reference period (same weeks in 2018 and 2019), correlating with state-specific social distancing index (SDI).

**Results:**

EPLB and EPIS percentages did not significantly decrease (1.58–1.45%, *p* = 0.07, and 0.08–0.06%, *p* = 0.14, respectively). SDI was not significantly correlated with percent change of EPLB (CC = 0.29, 95% CI = −0.12, 0.71) or EPIS (CC = −0.23, 95% CI = −0.65, 0.18). Percent change in mean gestational age was positively correlated with SDI (CC = 0.49, 95% CI = 0.07, 0.91).

**Conclusions:**

Increased social distancing was not associated with change in incidence of EPLB but was associated with a higher gestational age of extremely preterm births.

**ClinicalTrials.gov ID:**

Generic Database: NCT00063063.

## Introduction

The COVID-19 pandemic severely affected health and healthcare systems across the globe [1–4], with significant effects on healthcare availability [5, 6], health-seeking behavior [7–10], and outcomes of COVID-19 infected and non-infected patients [10–13]. The pandemic resulted in differently timed and variably implemented national and local governmental actions, including lockdowns and home quarantine requirements [14], and changes in public health behaviors, including social distancing, use of face coverings, and enhanced sanitary measures like frequent hand washing and use of hand sanitizers.

Observational studies published before the onset of the COVID-19 pandemic have reported associations between increased occupational physical activity [15–17], stress [18, 19], and infection [20] with an increase in the risk of preterm birth; however, the strength of evidence is low due to limited rigor and marked heterogeneity in interventions and outcome measurements. Pandemic-related governmental actions and changes in public health behaviors may have led to a lower incidence of extremely preterm live births associated with decreases in occupational physical activity, physical stress, and infections on a population level, providing a good investigative opportunity to fill this research gap. COVID-19 pandemic-associated overall changes in maternal and perinatal outcomes have been reported [21–25]. The studies reporting changes in the incidence of preterm births during the pandemic period [24–28] and correlations with the timing of lockdowns [29–32] show inconsistent findings. The majority of these studies have defined preterm birth as birth <37 weeks gestational age, and none, to our knowledge, have focused on extremely preterm births (gestational ages from 22^0/7^ to 28^6/7^ weeks). Given that extremely preterm births are responsible for substantial neonatal morbidity and mortality [33, 34], it is important to identify the effect of the pandemic on this population.

Variability associated with the pandemic related to the implementation, public adherence to lockdowns, and mobility restrictions provided an opportunity to objectively examine the changes in extremely preterm births with pandemic-related social distancing metrics. The aim of the current study was to test the hypothesis that increased social distancing was associated with a lower incidence of extremely preterm live births during the initial COVID-19 pandemic period using the large and diverse *Eunice Kennedy Shriver* National Institute of Child Health and Human Development (NICHD) Neonatal Research Network (NRN) registries.

## Methods

### Study design and participants

This observational study used prospectively collected data from the All Birth Cohort database and Generic Database of the NICHD NRN. The NRN sites (listed in the appendix) are academic centers that are selected by the NICHD using a peer review process. The databases include outcomes of extremely preterm (gestational ages from 22^0/7^ to 28^6/7^ weeks) births, including intrapartum stillbirths (EPIS) and live births (EPLB) from the NRN sites. All participating hospital institutional review boards approved participation in the databases with or without waiver of consent [35]. We also retrospectively collected summary data on live births and stillbirths at gestational age ≥22^0/7^ weeks by reviewing labor and delivery records from the NRN sites for the corresponding study periods.

The study included consecutive births during the calendar weeks 9–30 from 3/1/2020 to 8/1/2020 (a period of 5 consecutive months) at 26 hospitals participating in the NRN across the United States as the pandemic period and births in the corresponding calendar weeks of 2018 and 2019 as the reference period. The study period was selected to include the pandemic period of lockdowns and mobility restrictions in the United States and the adjacent periods of unrestricted mobility for assessing the correlation of change between social distancing and outcomes [36, 37]. The comparison with corresponding calendar weeks of 2018 and 2019 in the reference period was chosen to avoid confounding by possible seasonal trends for preterm births [38–40].

## Outcomes

The primary outcome was the proportion of EPLB among all live births. The secondary outcomes were the proportion of EPIS among all births and the correlation of the social distancing index (SDI) with the percent change of EPLB and EPIS.

## Comparison

The proportions of EPLB and EPIS during the pandemic period were compared to rates during the years 2018 and 2019. The denominator used to calculate the proportion of EPLB was live births, and that for EPIS was total births (stillbirths + live births). Maternal and fetal-neonatal characteristics and infant outcomes (immediate postnatal outcomes and hospital outcomes by 120 days after birth) were compared between the reference and the pandemic period.

## Definitions

Calendar weeks were defined as the first week starting from the first Sunday of the year. Live birth was defined as presence of heart beats at birth. A complete course of antenatal steroids was defined as a course of 2 doses of betamethasone or 4 doses of dexamethasone, with at least 24 h between the first dose and delivery. Antepartum bleeding was defined as the presence of placenta previa, abruption, or threatened abortion resulting in external or occult bleeding after 20 weeks of pregnancy, not including bloody show. The race was categorized as Black, White, or Other based on self-reported maternal race. Similarly, ethnicity was categorized as Hispanic-Latino or Other based on self-reported maternal ethnicity. Insurance status was determined based on maternal medical insurance. Public insurance was defined as insurance by Medicaid, Medicare, a state-funded program, a federally funded program, or insurance obtained through the Affordable Care Act. Private insurance was defined as traditional insurance or managed care (including CHAMPUS, TRICARE, or any insurance that may be tied to work). Self-Pay/uninsured was defined as hospitalization expenses paid for by the mother or other responsible party. Gestational age was defined by the best estimate of gestational age in weeks and days, using obstetrical measures based on the last menstrual period, obstetrical parameters, and/or early (first trimester) prenatal ultrasound as recorded in the maternal chart, and when these were unavailable or considered unreliable, the neonatologist’s estimate based on the neonatal exam (Ballard or Dubowitz) was used. Intraventricular hemorrhage (IVH) was defined using Papile classification, with grade 3 IVH defined as IVH with ventricular dilation and grade 4 IVH defined as IVH with parenchymal hemorrhage [41]. Proven necrotizing enterocolitis was defined as stage 2 or above using the modified Bell’s staging criteria [42]. SDI was defined as the extent to which residents of the state practiced social distancing, computed from six mobility metrics (including % staying home, % reduction of all trips compared to pre-COVID-19 benchmark, % reduction of work trips, % reduction of non-work trips, % reduction of travel distance, and % reduction of out-of-county trips, available from https://data.covid.umd.edu). The social distancing index used in the current manuscript was chosen as it was from a publicly available research database from the University of Maryland and was generated with robust data processes and methods [43].

## Statistical analysis

Statistical significance for unadjusted baseline and outcomes comparisons was determined using Mann–Whitney, Fisher exact, or chi-square tests. Incidence rates of EPLB and EPIS were calculated for weeks 9–30 of each time period (reference and pandemic periods) and weekly outcome comparisons were performed. Pre-trends in the rates of EPLB and EPIS leading into the pandemic were assessed using an interrupted time-series (ITS) analysis. For the 2-year reference period, incidence rates were averaged within each calendar week.

LOESS-smoothed time series of the percent change from the reference period of the incidence of EPLB and EPIS with SDI and mean gestational age with SDI were plotted for weeks 9–30. Analysis of cross-correlation [44] was done for the change in the incidence of EPLB and EPIS over time with the social distancing as measured by site state-specific SDI. Lags in the correlations with SDI up to 5 weeks for EPLB, EPIS, and gestational age were examined. Measures of the outcome incidences and the SDI were weighted by population and summed across sites to get overall measures to use in the cross-correlation analysis. A 2-sided *P* value of less than .05 was used to define statistical significance. All comparisons were considered exploratory, and no adjustments for multiple comparisons were made. The study is reported as per the STROBE (Strengthening the Reporting of Observational Studies in Epidemiology) statement for reporting observational studies [45].

## Results

There were 133,185 births during the study period, of which 89,409 were in the reference period (calendar weeks 9–30 of 2018 and 2019), and 43,776 were in the pandemic period (calendar weeks 9–30 of 2020). There were 2036 EPLB and 97 EPIS, of which 1405 and 72 were in the reference period, and 631 and 25 were in the pandemic period, respectively (Table 1). The proportions of EPLB and EPIS did not significantly decrease in the pandemic period (1.58 to 1.45%, *p* = 0.07, and 0.08 to 0.06%, *p* = 0.14, respectively). Analysis of pre-trends indicated no significant increase or decrease in the rates of EPLB and EPIS between the time leading up to the pandemic and during the pandemic (*p* = 0.80).

**Table1:** Baseline characteristics and outcomes.

Extremely preterm intrapartum stillbirths					
Characteristic	Category	Reference period (*N* = 72)	Pandemic period (*N* = 25)	Mean difference/odds ratio (95% CI)	*P*-value
Antenatal steroids, *n*/*N* (%)	Complete course	17/72 (23.6)	9/24 (37.5)	ref.	0.27
	Partial course	10/72 (13.9)	1/24 (4.2)	0.20 (0.00, 1.81)	
	None	45/72 (62.5)	14/24 (58.3)	0.59 (0.19, 1.85)	
Antepartum bleeding, *n*/*N* (%)		19/72 (26.4)	9/24 (37.5)	1.66 (0.55, 4.90)	0.44
Race, *n*/*N* (%)	Black	32/70 (45.7)	13/24 (54.2)	ref.	0.70
	White	33/70 (47.1)	10/24 (41.7)	0.75 (0.25, 2.15)	
	Other	5/70 (7.1)	1/24 (4.2)	0.50 (0.01, 5.12)	
Ethnicity, *n*/*N* (%)	Hispanic- Latino	12/71 (16.9)	7/24 (29.2)	0.50 (0.15, 1.74)	0.24
	Other	59/71 (83.1)	17/24 (70.8)	ref.	
Insurance, *n*/*N* (%)	Public	39/71 (54.9)	18/24 (75.0)	0.41 (0.12, 1.24)	0.10
	Private	32/71 (45.1)	6/24 (25.0)	ref.	
Gestational age (weeks), M (SD)		23.6 (1.7)	24.1 (1.9)	−0.5 (−1.4, 0.4)	0.29
Birth weight (g), M (SD)		517.6 (260.3)	490.9 (334.2)	26.7 (−137.0, 190.3)	0.74
**Extremely Preterm Live births**					

Characteristic	Category	Reference period (*N* = 1405)	Pandemic period (*N* = 631)	Mean difference/odds ratio (95% CI)	*P*-value
Antenatal steroids, *n*/*N* (%)	Complete course	971/1403 (69.2)	398/628 (63.4)	ref.	0.012
	Partial course	299/1403 (21.3)	171/628 (27.2)	1.40 (1.11, 1.75)	
	None	133/1403 (9.5)	59/628 (9.4)	1.08 (0.77, 1.52)	
Antepartum bleeding, *n*/*N* (%)		302/1405 (21.5)	172/631 (27.3)	1.37 (1.09, 1.71)	<0.01
Race, *n*/*N* (%)	Black	550/1349 (40.8)	265/596 (44.5)	ref.	0.30
	White	715/1349 (53.0)	298/596 (50.0)	0.87 (0.71, 1.06)	
	Other	84/1349 (6.2)	33/596 (5.5)	0.82 (0.51, 1.27)	
Ethnicity, *n*/*N* (%)	Hispanic- Latino	205/1384 (14.8)	105/616 (17.0)	1.18 (0.90, 1.54)	0.20
	Other	1179/1384 (85.2)	511/616 (83.0)	ref.	
Insurance, *n*/*N* (%)	Public	743/1400 (53.1)	337/629 (53.6)	ref.	0.017
	Private	621/1400 (44.4)	261/629 (41.5)	0.93 (0.76, 1.13)	
	Self- pay/uninsured	36/1400 (2.6)	31/629 (4.9)	1.90 (1.11, 3.21)	
Gestational age (weeks), M (SD)		26.1 (1.9)	26.2 (1.8)	−0.1 (−0.3, 0.1)	0.19
Birth weight (g), M (SD)		825.7 (264.9)	846.1 (259.9)	−20.4 (−45.0, 4.2)	0.10
**Hospital outcomes**					
Death <12 h after birth, *n*/*N* (%)^a^		106/1405 (7.5)	19/631 (3.0)	0.38 (0.22, 0.63)	<0.01
				0.35 (0.20, 0.63)	<0.01
Discharge home, *n*/*N* (%)^a^		1054/1405 (75.0)	497/627 (79.3)	1.27 (1.01, 1.61)	0.042
				1.23 (0.95, 1.60)	0.11
Steroids for bronchopulmonary dysplasia, *n*/*N* (%)^a^		328/1296 (25.3)	143/608 (23.5)	0.91 (0.72, 1.14)	0.43
				0.92 (0.73, 1.17)	0.51
Intraventricular hemorrhage (grade 3 or 4), *n*/*N* (%)^a^		220/1264 (17.4)	80/591 (13.5)	0.74 (0.56, 0.99)	0.036
				0.65 (0.48, 0.87)	<0.01
Proven necrotizing enterocolitis, *n*/*N* (%)^a^		132/1295 (10.2)	58/607 (9.6)	0.93 (0.66, 1.30)	0.68
				0.92 (0.66, 1.28)	0.64
Late-onset culture positive septicemia, *n* (%)		248/1255 (19.8)	107/580 (18.4)	0.92 (0.71, 1.19)	0.53
				0.92 (0.71, 1.20)	0.55

^a^Second row of odds ratios are adjusted for sex, gestational age, birth weight, multiple births, and antenatal steroids.

**Fig. 1:**
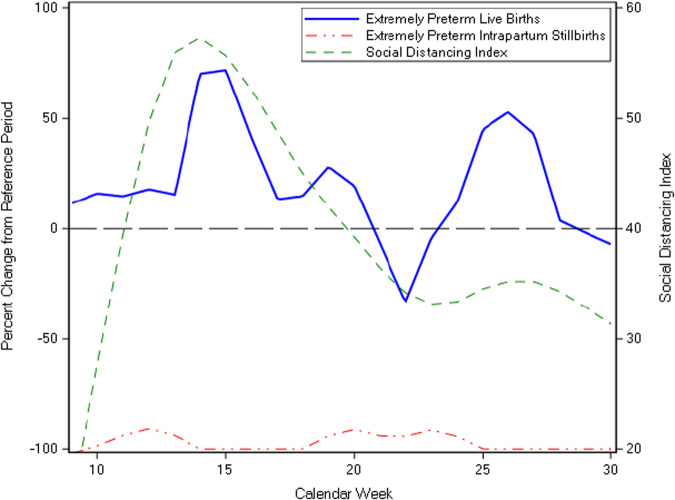
Correlation of social distancing index with percent change in extremely preterm live births and intrapartum stillbirths. The percent change from reference period ((pandemic – reference)/reference*100) was calculated using the weekly rates of EPLB and EPIS. The social distancing index was not significantly correlated with the percent change of extremely preterm live births (CC = 0.29, 95% CI = −0.12, 0.71) and intrapartum stillbirths (CC = −0.23, 95% CI = −0.65, 0.18). Reference period: calendar weeks 9–30 of 2018 and 2019 pandemic period: calendar weeks 9–30 of 2020.

**Fig. 2:**
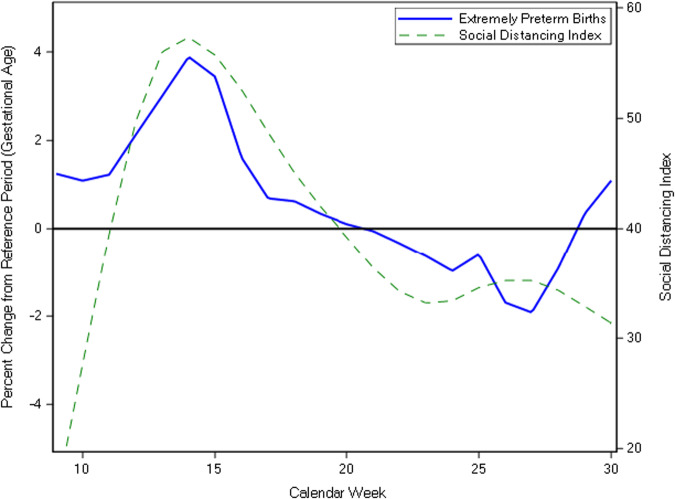
Correlation of social distancing index with percent change in extremely preterm birth gestational age. The percent change from reference period ((pandemic – reference)/reference*100) was calculated using the weekly average gestational age. The social distancing index was positively correlated with extremely preterm birth gestational age (CC = 0.49, 95% CI = 0.07, 0.91). Reference period: calendar weeks 9–30 of 2018 and 2019 pandemic period: calendar weeks 9–30 of 2020.

The SDI (mean ± SD = 39.9 ± 11.3 for the pandemic period) was not significantly correlated with the percent change of EPLB (cross-correlation coefficient, CC = 0.29, 95% CI = −0.12, 0.71). We also analyzed the correlation of SDI with outcomes, introducing weekly lag periods, and found that for the outcome of percent change of EPLB there was no increase in correlation coefficient with the addition of lag periods (Supplementary Table 1). The SDI was also not significantly correlated with the percent change in EPIS (CC = -0.09, 95% CI = −0.32, 0.51, lag of 1 week, Fig. 1, Supplementary Table 1). In the pandemic period, several weeks (8/22) recorded no EPIS cases (Supplementary Table 2). For the first half of the study period (weeks 9–18) the mean gestational age for all extremely preterm births was higher in the pandemic period compared to the reference period (mean ± SD = 26.2 ± 1.8 vs. 25.8 ± 1.9 weeks, *p* < 0.01). The percent change in mean gestational age for all preterm births was positively correlated with the SDI (CC = 0.49, 95% CI = 0.07, 0.91, lag of 0 weeks, Fig. 2, Supplementary Table 1), however, the mean gestational age for infants born during weeks 9–30 was not significantly different between the study periods (Table 1).

Baseline characteristics differed between the two epochs among EPLB and EPIS. For example, in the EPLB group, during the pandemic period, higher rates of antenatal bleeding (21.5–27.3%, OR = 1.37, 95% CI = 1.09, 1.71, *p* < 0.01), higher rates of self-pay/uninsured (2.6–4.9%, OR = 1.90, 95% CI = 1.11, 3.21, *p* = 0.017), and higher rates of partial antenatal steroid course (21.3–27.2%, OR = 1.40, 95% CI = 1.11, 1.75, *p* = 0.012) were noted (Table 1). Death <12 h after birth and IVH grade 3 or 4 were significantly lower in the pandemic period, adjusting for sex, gestational age, birth weight, multiple births, and antenatal steroids. On unadjusted analysis, discharge home was significantly higher in the pandemic period, but it was not significantly different on adjusted analysis. Steroids use for bronchopulmonary dysplasia, late-onset culture positive septicemia, and proven necrotizing enterocolitis were lower during the pandemic period compared to the reference period but were not statistically significant (Table 1).

## Discussion

This study from 26 US hospitals in the NICHD Neonatal Research Network tested the hypothesis that increased social distancing was associated with a lower incidence of extremely preterm live births during the initial COVID-19 pandemic period. We found that increased social distancing was not associated with a change in the incidence of extremely preterm live birth or intrapartum stillbirth but was associated with a slightly higher gestational age of the extremely preterm births in the initial COVID-19 pandemic period. The rates of EPIS were low in both periods making it difficult to make statistical inferences. There were higher rates of antenatal bleeding, self-pay/uninsured, and partial course of antenatal steroid in the pandemic period; these changes could reflect pandemic-related healthcare disruptions, including the social distancing measures or other unaccounted factors that are listed in the limitations section. Despite these baseline differences, there was a decrease in adjusted rates of death <12 h after birth and IVH grade 3 or 4 for infants in the pandemic period, possibly related to an increase in mean gestational age for all preterm births in the pandemic period.

Published studies have primarily compared preterm birth rates in the pandemic period with the prior period without adjusting for pandemic-related metrics; the majority of them have been single-center studies [25, 46]. Multicenter and regional studies correlating the time of the lockdowns with preterm births have shown inconsistent findings, varying from a decrease [47–49], no change [50, 51], to an increase in the rates of preterm births [52], possibly due to variations in social distancing and other public health measures. To our knowledge, the current study is the first to report changes in the incidence and outcomes of extremely preterm births and analyze the correlation of extremely preterm births and outcomes with the state-specific social distancing index, a composite metric of several pandemic-related, population-based, objective social distancing and mobility metrics. The current study expands on the previous findings to inform the pandemic-related pregnancy and perinatal public health policy. The strong temporal correlation, especially in the early pandemic period, between the social distancing index and extremely preterm birth gestational age should be prospectively evaluated. We speculate that the improvement in the hospital outcomes of extremely preterm infants seen in the current study may have been due to infection prevention measures such as hand hygiene, visitor restriction, and universal masking of care providers instituted during the pandemic period, a hypothesis that deserves further testing.

The current study leverages high-quality prospective research databases of the NRN with quality control and ascertainment of births and fetal-neonatal/infant outcomes. The study is based on data from 26 hospitals across the US and, due to diversity in geography and implementation of pandemic-related policies, provides an opportunity to address the knowledge gap regarding the effect of pandemic-related social distancing on the incidence of preterm births. The present study was able to leverage more detailed information on practices such as antenatal steroids and specific neonatal morbidities commonly associated with prematurity by utilizing the NRN database, which is not accessible through population registries based on data from birth and death certificates.

However, there are a few limitations that need to be considered. The study cohort does not represent all births in a well-defined geography. It is possible, for example, that the decrease in extremely preterm births observed resulted from changes in hospital referral patterns or an increased rate of antepartum stillbirth. The registries used for this study are based on the deliveries in participating hospitals. Additionally, the SDI was measured at the state level, and specific practices may have varied within states or among hospitals or hospital-referral populations. Also, this analysis could not account for additional factors that may have potentially contributed towards the observed outcome differences, including factors such as decreased access to care, health seeking behavior changes leading to delay in care, decreased access to pregnancy termination services, and changes in insurance status due to employment disruptions.

## Conclusions

Increased social distancing was not associated with a change in the incidence of extremely preterm live birth in a cohort of US academic medical centers but was associated with a higher gestational age of the extremely preterm births in the initial COVID-19 pandemic period.

### Access to data and data analysis

BC and AD had full access to all the data in the study and take responsibility for the integrity of the data and the accuracy of the data analysis.

### Supplementary information

Supplemental Material

## Data Availability

Data reported in this paper may be requested through a data use agreement. Further details are available at https://neonatal.rti.org/index.cfm?fuseaction=DataRequest.Home.
