# Supplementary material for: Social distancing and extremely preterm births in the initial COVID-19 pandemic period
Source: J Perinatol. Author manuscript; Available in PMC 2025 Jul 1. (PMC11226398; doi:10.1038/s41372-024-01898-3)
Supplement: Supplementary data [file NIHMS1971406-supplement-Supplementary_data.pdf]

Supplementary Table 1: Cross-Correlation of Social Distancing Index with Percent Change of EPLB, EPIS, and Gestational Age

Lag (Weeks)	CC	95% CI
**EPLB**		
**0**	**0.29**	**-0.12, 0.71**
1	0.21	-0.21, 0.62
2	0.08	-0.34, 0.49
3	0	-0.42, 0.42
4	-0.02	-0.44, 0.4
5	0	-0.42, 0.42
**EPIS**		
0	0.08	-0.34, 0.50
**1**	**0.09**	**-0.32, 0.51**
2	0.01	-0.41, 0.43
3	0.01	-0.41, 0.42
4	0	-0.41, 0.42
5	-0.03	-0.45, 0.38
**Gestational Age**		
**0**	**0.49**	**0.07, 0.91**
1	0.42	0.00, 0.84
2	0.41	-0.01, 0.83
3	0.41	-0.01, 0.82
4	0.23	-0.19, 0.64
5	0.16	-0.25, 0.58

Supplementary Table 2: Distribution of Extremely Preterm Live Births and Intrapartum Stillbirths by Study Period*

Study Period	Reference Period						Pandemic Period					
**Week Number**	**Total Live Births**	**Total Stillbirths**	**Number of EPLB**	**Numbe r of EPIS**	**EPLB %**	**EPIS %**	**Total Live Births**	**Total Stillbirths**	**Number of EPLB**	**Number of EPIS**	**EPLB %**	**EPIS %**
9	3905	42	62	5	1.59%	0.13%	1975	20	33	2	1.67%	0.10%
10	3835	27	62	3	1.62%	0.08%	1941	13	21	1	1.08%	0.05%
11	4050	51	62	3	1.53%	0.07%	1909	10	33	3	1.73%	0.16%
12	3918	35	81	4	2.07%	0.10%	1941	15	18	2	0.93%	0.10%
13	3984	28	58	3	1.46%	0.07%	1922	15	40	0	2.08%	0.00%
14	3941	34	76	4	1.93%	0.10%	1914	17	26	0	1.36%	0.00%
15	3856	37	55	2	1.43%	0.05%	1881	24	32	0	1.70%	0.00%
16	3910	30	65	4	1.66%	0.10%	1924	15	26	2	1.35%	0.10%
17	3954	39	54	5	1.37%	0.13%	1967	19	27	2	1.37%	0.10%
18	3989	29	68	3	1.70%	0.07%	1933	22	35	1	1.81%	0.05%
19	3983	32	62	3	1.56%	0.07%	1895	17	34	1	1.79%	0.05%
20	4128	37	63	3	1.53%	0.07%	1980	21	35	2	1.77%	0.10%
21	3976	33	57	6	1.43%	0.15%	1906	11	23	1	1.21%	0.05%
22	4148	37	63	5	1.52%	0.12%	1974	18	31	0	1.57%	0.00%
23	4158	39	65	4	1.56%	0.10%	1985	16	35	1	1.76%	0.05%
24	4070	33	59	2	1.45%	0.05%	1927	18	25	0	1.30%	0.00%
25	4158	30	78	3	1.88%	0.07%	1961	21	29	2	1.48%	0.10%
26	3932	31	69	2	1.75%	0.05%	1990	11	26	0	1.31%	0.00%
27	4239	39	59	4	1.39%	0.09%	2182	13	24	0	1.10%	0.00%
28	4104	36	78	1	1.90%	0.02%	2125	12	27	3	1.27%	0.14%
29	4197	37	55	2	1.31%	0.05%	2056	16	28	0	1.36%	0.00%
30	4216	22	54	1	1.28%	0.02%	2132	12	23	2	1.08%	0.09%

*Births in the Neonatal Research Network Hospitals. Reference period= calendar weeks 9 to 30 of 2018 and 2019, pandemic period=calendar weeks 9 to 30 of 2020, EPLB= extremely preterm live births, and EPIS= extremely preterm stillbirths.
